# Chloroform and Its Dangers

**Published:** 1849

**Authors:** D. Meredith Reese

**Affiliations:** New York


### ARTICLE VI.

### *Chloroform and its Dangers.*

*To the Editor of the Boston Medical and Surgical Journal.*

Dear Sir,—The death of a patient in the New York City Hospital by chloroform, within the past week, will very probably be disastrous to the profession and the public, by being perverted into an argument against all use of this invaluable anæsthetic agent, thus bringing into temporary disrepute one of the noblest dicoveries in the healing art, which has ever been developed. It is in this view only, that the case furnishes a theme for animadversion in the journals of the profession, for the possibility of so potent a remedy being fatal without great caution, must be obvious, and the same is true of morphine, prussic acid, and many other active agents which are in established and hab'tual use. Nor does the very rare occurrence of death from an accidental overdose of either of them, furnish any rational argument against their utility or safety, especially when it is known that some obscure idiosyncracy may account for such untoward results, even when active remedies are given with the utmost cantion, both as respects the dose and the pathological state existing at the time.

Still however the fatal case at New York should serve as an admonition, if any be needed, to the very discreet employment of chloroform by inhalation, at all times; but especially when, as in certain severe operations, its full effects is desired. Next to the judicious selaction of the cases in which to employ this agency, in relation to which the profession are agreed, two things are important: viz., 1st. Never to allow the patient to inhele chloroform, without ocasionally withdrawing it from the mouth and nostrils, thus permitting the inspiration of the atmospheric air at brief intervals; and this in view of the dangers of its continous inhalation even for two or three minutes; and 2d. Always to watch the effects upon the pulse, for the purpose of instantly desisting from its use on any considerable failure either in its force or frequency. With these precautions, there ueed be no apprehension of any fatal or mischievous results in any patient, of any age, whose morbid condition is unaccompanied by local lesions of some vital organ; in which case, however, the use of chloroform is containdicated. But without such precautions, this remedy, undulated, is liable to be so rapid and violent in its action as to endanger life. And from a conviction of its occasional hazards, and the possibility of its proving fatal as seen in the rare examples which have occured here and elsewhere, there are not a few surgeons who have obandoned this article, and prefer to rely upon the sulphuric and chloric ether, notwithstanding the slower and less perfect anæthesia which often results, and this even at the risk of occasional failure to suspend sensibility to the desired extent, by these weaker articles.

A still preferable course, however. is pursued by others, who are unwilling to deprive their patients of the superiority hey justly ascribe to chloroform, and hence they seek to remove its hazards by diluting it with ether, thus diminishing both its rapidity and violence, and as it is believed, after ample experience, annihilating all its dangers. The proportions of such dilution employed during the last year in the Bellvue Hospital, are equal parts by weight; or, what is the same thing, one part by chloroform to three or four parts of ether by *measure*. The mixture should be made extemporaneously at the time of using, as it is otherwise liable to deteriorate. The same precautions are discreet, which which have been advised in reference to the undiluted article, and with these, after extensive opportunities of witnessing the employment of this *mixture*, in surgical, medical, and obstetrical practice, including sevaral hundred cases, I am prepared to testify that no untoward result has followed in a single instance. So complete is the insensibility which has been produced that in one example of amputation of the thigh, several days elapsed before the patient discovered that he had lost a limb; and he betrayed the most unaffected surprise when he realized the mutilation he had suffered, the fact being revealed to him for the first time when it was proposed to dress the stump, which became necessary on the fifth day after the operation. In every case the insensibility to pain has been compleie, and this state has been perpetuated, by the occasional repetition of the dose, when necessary, as in protracted operations. The quantity found sufficient has varied from half an ounce to two or three ounces of the mixture, and applied to the nostrils and mouth by a towel or sponge. In some instances the inhalation for four or five minutes has produced the full effect, as indicated by approaching stertor, with muscular relaxation; but in other examples double and even triple this time has been called for. The effect usually passes off, and full conciousness returns, in from three to five minutes after the inhalation ceases; but this result may be hastened by sprinkling cold water upon the face. No unfavorable consequences, even of temporary character have been observed to follow the inhalation, and it is rare that either coughing or nausea occurs to interrupt the prcess.

I regard it so imporant that no prejudice should be produced against the use of anæsthetic agents, by the recent disaster at the City Hospital, that I have written thus much in their defence, and at the same time suggested what I conceive to be the salutary and necessary precautions which the profession should adopt if the would disabuse they public mind, and protect these valuable remedies from being thrown into disrepute. If you concur with me in opinion that these brief hints may be useful at this crisis, I may find time to communicate more fully on the subject.

Respectfully yours,

D. MEREDITH REESE.

*New York, Jan. 22nd,* 1849.

[We fully agree with our respected correspondent as to the injury that would accrue, both to the public and the profession, from the use of anæsthetic agents falling into disrepute on account of the occasional unfavorable results attending their administration. Every well attested means, therefore, for preventing these results, should be adopted. That recommended by Dr, R., in the above communication, and which we trust he will soon treat further, appeas judicious, and having the recommendation of his experience and approval, is certainly worthy the trial of others.—ED.]—*Bost. Med. and Surg. Jour.*

